# Flexible wearable sensors for crop monitoring: a review

**DOI:** 10.3389/fpls.2024.1406074

**Published:** 2024-05-29

**Authors:** Baoping Yan, Fu Zhang, Mengyao Wang, Yakun Zhang, Sanling Fu

**Affiliations:** ^1^ College of Agricultural Equipment Engineering, Henan University of Science and Technology, Luoyang, China; ^2^ College of Physical Engineering, Henan University of Science and Technology, Luoyang, China

**Keywords:** crop monitoring, sensor technology, flexible wearable sensor, smart agriculture, food security

## Abstract

Crops were the main source of human food, which have met the increasingly diversified demand of consumers. Sensors were used to monitor crop phenotypes and environmental information in real time, which will provide a theoretical reference for optimizing crop growth environment, resisting biotic and abiotic stresses, and improve crop yield. Compared with non-contact monitoring methods such as optical imaging and remote sensing, wearable sensing technology had higher time and spatial resolution. However, the existing crop sensors were mainly rigid mechanical structures, which were easy to cause damage to crop organs, and there were still challenges in terms of accuracy and biosafety. Emerging flexible sensors had attracted wide attention in the field of crop phenotype monitoring due to their excellent mechanical properties and biocompatibility. The article introduced the key technologies involved in the preparation of flexible wearable sensors from the aspects of flexible preparation materials and advanced preparation processes. The monitoring function of flexible sensors in crop growth was highlighted, including the monitoring of crop nutrient, physiological, ecological and growth environment information. The monitoring principle, performance together with pros and cons of each sensor were analyzed. Furthermore, the future opportunities and challenges of flexible wearable devices in crop monitoring were discussed in detail from the aspects of new sensing theory, sensing materials, sensing structures, wireless power supply technology and agricultural sensor network, which will provide reference for smart agricultural management system based on crop flexible sensors, and realize efficient management of agricultural production and resources.

## 1 Introduction

Food security became the focus of global concern, which was particularly crucial to ensure the safety of people’s lives. According to the Global Food Crisis Report 2023, the number of people facing sudden and severe food insecurity and in urgent need of food, nutrition and livelihood assistance in 2022 had increased for four consecutive years, more than 250 million people were facing severe hunger, and people in seven countries were on the verge of famine (WFP, 2023). As the main source of human food, crops met the increasingly diversified consumer demand, which was crucial to human food supply and food security (Zhang et al., 2023a). Stresses in crops can be broadly separated into biotic stress, which is stress caused by living organisms such as pathogens or herbivores, and abiotic stress, which is stress caused by environmental factors. Both types of stresses can lead to reduced growth, damage and death of crops, impacting yields (Coatsworth et al., 2023). Therefore, exploring crop growth mechanism and stress response are particularly important. Considering that the growth and development of crops and their responses to external stimuli are very slow and subtle, long-term monitoring of crop phenotypes is required to better understand their growth and the mechanism with the surrounding environment (Bahuguna and Jagadish, 2015).

Crop phenotype refers to the physical, physiological and biochemical characteristics, which reflects the structure and function of crop cells, tissues, organs, plants, populations, including nutrient, physiological and ecological information (Dejonghe and Russinova, 2017). Nutrient information mainly includes nitrogen, phosphorus, potassium and other substances related to crop physiological activities, which can be extended to water content and chlorophyll. Physiological information of crops contains proteins, nitrogen, alcohols, anti-oxidation and other functional indicators under normal growth, biotic stresses such as diseases, pests, and weeds, and abiotic stresses such as cold, high temperature, and salinity. Ecological information refers to the external physical information of crops to the growth environment, such as leaf thickness, fruit size, stem diameter, stem flow rate, root hydraulic conductivity (Busby et al., 2017).

In precision agriculture, sensors have been widely used to monitor crop health and growth, providing key information for optimizing crop growth environment and combating biotic and abiotic stresses. Wearable crop devices can be placed and attached to different parts of the crop (such as roots, stems or leaves), using a series of physical, chemical and biological sensors to extract physiological (biophysical or biochemical) information in real time in a non-invasive or minimally invasive manner to achieve on-site monitoring of crop health status (Zhao et al., 2020) (Zhou et al., 2024). However, the existing crop sensors were mainly rigid mechanical structures, which would cause biological rejection and damage the organs while contact with crop tissues and organs for a long time (Aroca et al., 2016; Moreno-Moreno et al., 2018). It is difficult to meet the long-term and continuous monitoring needs of crop physiological information. Flexible sensors refer to electronic devices made of flexible materials, which have good flexibility, ductility, and can be freely bent or even folded. In order to realize non-destructive monitoring of crop phenotypes, researchers have tried to wear flexible electronic sensors for crops. The mechanical properties of flexible sensors are similar to crop tissues. And the flexible sensors can be integrated with crops without additional rigid mechanical structures due to their excellent flexibility, ductility and biocompatibility (Qu et al., 2023). Not only the internal physiological information of crops but also the microenvironment information that directly affects crop phenotypes are monitored by flexible sensors, which will provide more real and intuitive data feedback for agricultural management and make effective decisions that are conducive to agricultural production.

**Figure 1:**
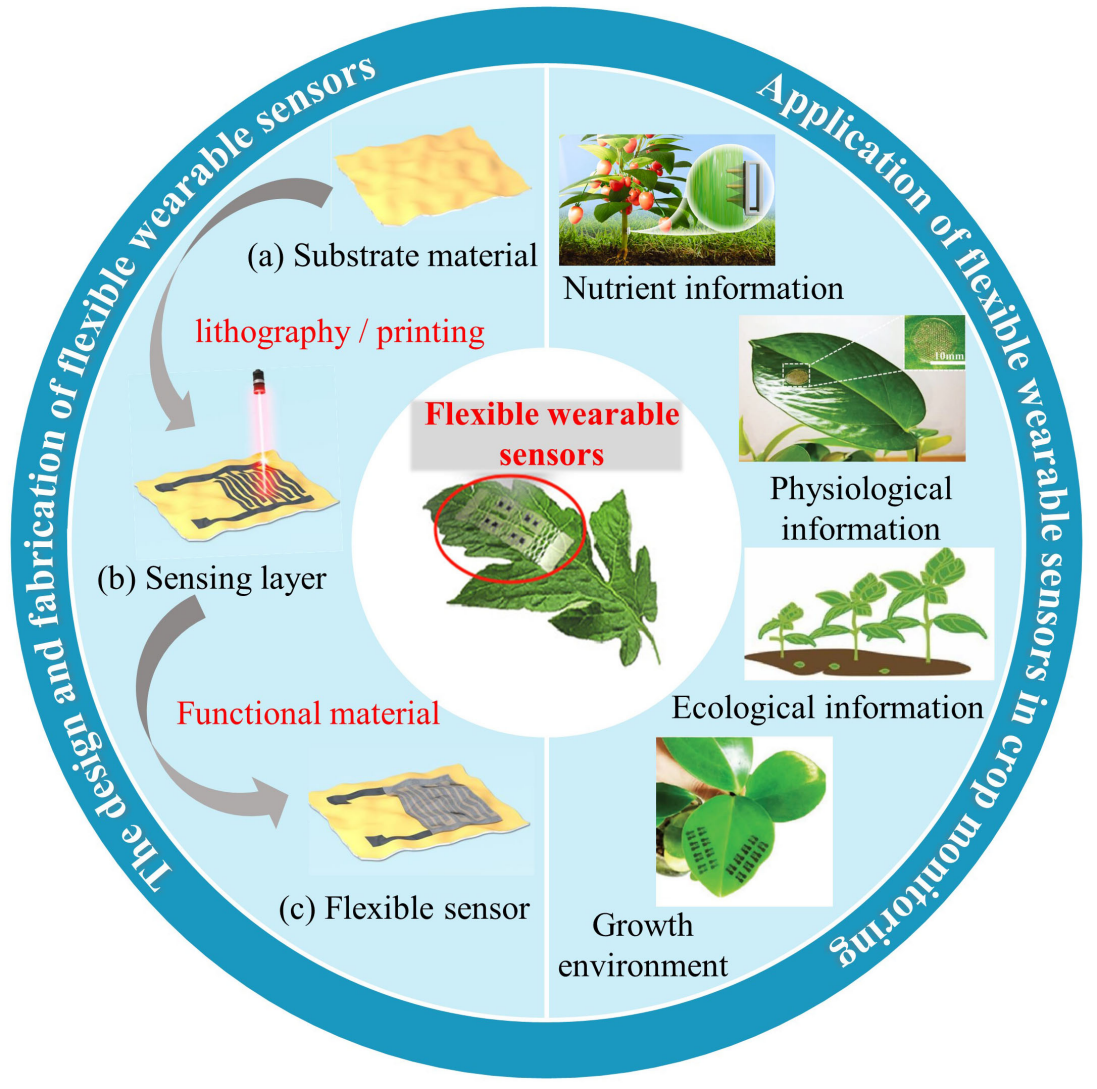
The significant process of crop wearables for smart agriculture including sensor design, fabrication (Lan et al., 2020) and application.

Based on the merits of flexible electronic devices, **Figure 1** outlines the design, fabrication, and application scenarios of crop wearables in smart agriculture. After summarizing the advantages and disadvantages of existing crop monitoring technologies, this review focuses on the preparation of crop flexible sensors and their monitoring roles in crop growth, such as monitoring crop nutrient, physiological, ecological information and growth environment. Finally, the problems and prospects of flexible wearable devices in crop monitoring are discussed in detail from the aspects of new sensing theory, sensing materials, sensing structures, wireless power supply technology and agricultural sensor network.

## 2 Advances of typical crop monitoring methods

Crop monitoring methods are mainly divided into non-contact and contact methods. The former such as optical imaging (Feng et al., 2021) and remote sensing (Lee et al., 2021) mostly predict the growth and development of crops by monitoring natural conditions and crop leaves, fruits, rhizomes, etc., providing scientific data for agricultural production to a certain extent. Compared with non-contact monitoring methods, wearable sensor has higher time resolution and spatial resolution, which use a mechanical clamping method to fix the sensor on the crop, and directly monitors the growth and growth microenvironment of the crop (Wang et al., 2018).

**Figure 2:**
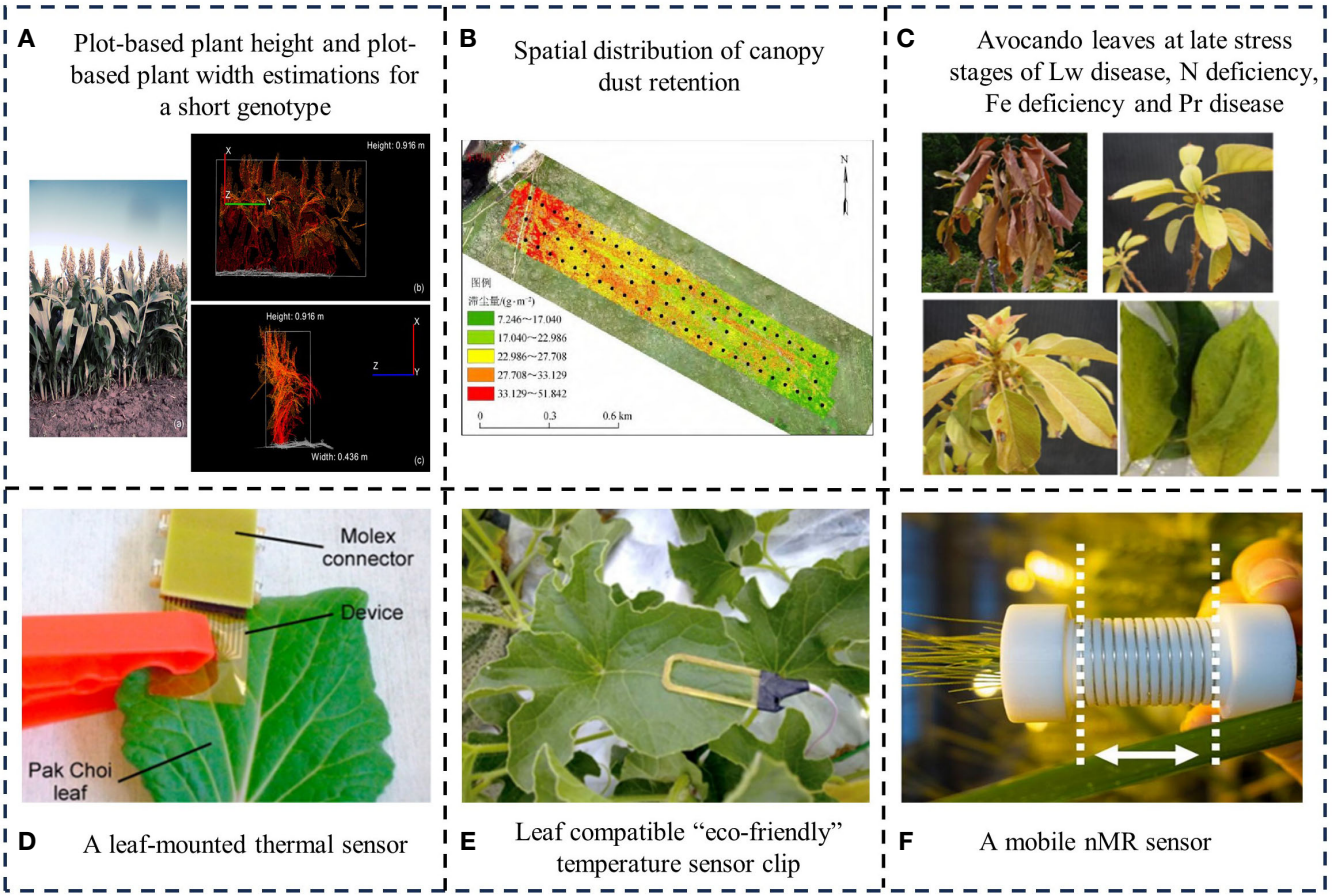
Application examples of typical crop monitoring methods.

Optical imaging is a monitoring method that uses optical sensors to observe and analyze crop phenotypes (Li et al., 2014). Wang et al. (2018) combined chlorophyll fluorescence imaging and multi-spectral imaging technology to distinguish common crop stress types such as drought, nutrient deficiency, and disease by monitoring tomato physiological information, providing the basis for automatic machine diagnosis. Mu et al. (2018) used drones to take high-resolution images of peach crowns, to analyze the crown width and crown project area of peach trees, and monitor the growth trend of orchard trees. Traditional optical imaging technology cannot obtain three-dimensional structure information of crops. To solve this problem, scholars have introduced 3D imaging technology. Bao et al. (2019) established a high-throughput phenotypic analysis system in the field, using multiple stereo cameras to photograph plants at the same time to quantify structures such as height, plant width, convex volume, surface area and stem diameter, so as to realize the structural characterization of dense-crown sorghum plants (**Figure 2A**). Jay et al. (2015) obtained RGB images by translating a single camera, and motion structure was used to establish a three-dimensional structure model of crop rows, characterizing crop plant height and leaf area. Based on color and height information, plants and backgrounds were distinguished, and the three-dimensional model scaling function was realized. It should be noted that such studies have high environmental requirements, the results were susceptible to light and background, resulting in poor monitoring stability.

By measuring the electromagnetic waves emitted or reflected by crops and using drone platforms equipped with multispectral, hyperspectral, thermal imaging, fluorescence and laser sensors, remote sensing technology can not only obtain a variety of crop information with dynamic changes in time and space, including crop type, physical characteristics (such as crop canopy temperature), chemical composition (such as leaf nitrogen content), structural characteristics (such as leaf inclination) and geometric characteristics (such as crop density), but also early diagnosis of crops diseases and insect pests, which provides scientific guidance for the effective management of crop diseases and insect pests. The dust retention of plant canopy was measured by the balance and leaf area meter, and the hyperspectral images were collected by airborne imaging spectrometer (Yan et al., 2022). The influence of dust retention on canopy spectral reflectance and trilateral parameters was analyzed, and the dust retention estimation model of plant canopy based on spectral parameters was established to realize the estimation of dust retention in grassland plant canopy based on UAV hyperspectral remote sensing (**Figure 2B**). Abdulridha et al. (2018) used remote sensing technology to detect avocado laurel wilt disease (**Figure 2C**) under stresses, and the automatically distinguish of healthy trees, trees infected with Phytophthora root rot, and trees under iron and nitrogen stresses was realized. When using remote sensing technology to monitor crops, due to the interference of environmental factors such as noise, light, wind, and soil during the flight of the drone, there are errors in the monitoring data, and it is difficult to obtain an accurate model. The crop data collected by the drone has gone through different growth stages of the crop. Therefore, the time-domain factors have a greater impact on the measurement of crop parameters, and crop information cannot be monitored in real time.

In order to realize real-time, high-throughput and high-resolution monitoring of crop phenotypic information, a mechanical device made of rigid material is used to fix the sensor on the crop. Atherton et al. (2012) proposed a leaf water content sensor, which was composed of a thin film microheater and two thin film thermocouples (**Figure 2D**). The two thermocouples recorded the temperature difference of the leaves caused by the heater and can be installed on the surface of the leaves to evaluate the water content of the leaves. The sensor integrated with the leaf surface by means of a plastic fixture based on a polyimide film with a thickness of 10μm. Palazzari et al. (2017) designed a clamping leaf sensor to monitor the leaf-air temperature difference closely related to water stress, and constructed a wireless sensor network by deploying several leaf sensors and soil moisture sensors in the field to provide data support for the automatic irrigation system of farmland (**Figure 2E**). Windt et al. (2021) used a small nuclear magnetic resonance instrument to monitor the water content of rice continuously and non-destructively under osmotic stress, as well as the dynamic accumulation of water and dry matter content during the development of wheat (**Figure 2F**). Such wearable sensors require additional mechanical structure to integrate with crops, and the mechanical structure is made of rigid hard materials. Its mechanical and physical properties are imcompatible with soft and deformable crops. Wearing sensors for a long time not only causes irreversible damage to crops, but also easily initiates crops self-healing mechanism, causing data distortion.

The emergence of flexible electronic devices provides new ideas for the field of crop monitoring. Flexible wearable sensors can be attached to crop organs without additional mechanical equipment, and have excellent flexibility, extensibility and biocompatibility, which solves the problem of incompatibility between traditional wearable sensors and vulnerable parts of crops. In addition, multi-functional flexible wearable devices can even detect multiple indicators at the same time, which can not only monitor crop growth, but also reflect climate change. The real-time, non-destructive and continuous *in-situ* monitoring of crop growth status, physiological or biochemical parameters is realized.

## 3 Manufacturing of the flexible sensors

The compatibility of flexible wearable sensors and crop organs should be considered to realize non-destructive real-time monitoring of crop phenotypic information. New flexible materials and advanced manufacturing processes will provide unlimited possibilities for the development of flexible wearable sensors.

### 3.1 Materials for flexible electronics

Several commonly used flexible materials are divided into three categories: substrate materials, functional materials and packaging materials according to specific applications as shown in **Table 1**.

**Table 1:** Materials for flexible electronics.

Category	Materials	Properties	Application
**Substrate/Packaging materials**	PDMS	Scalability, flexibility, transmittancy, gas permeability, adhesivity	Flexible substrate, sensor protection layer
	Eco-flex	Scalability, flexibility, degradability, transmittancy, gas permeability, adhesivity	Flexible substrate
	PU	Scalability, flexibility, transmittancy, gas permeability, adhesivity	Flexible substrate
	Aerogel	Scalability, flexibility, transmittancy, gas permeability, adhesivity	Flexible substrate
	Hydrogel	Scalability, flexibility, degradability, transmittancy, gas permeability, adhesivity	Flexible substrate, packaging
	PI	Scalability, flexibility, transmittancy, adhesivity	Flexible substrate, packaging, sensor protection layer
**Functional materials**	Metal nanometer	Conductivity, solubility	Flexible circuit
	Liquid metal	Conductive, low viscosity, excellent surface tension and biosafety	Flexible circuit
	Flexible conductive polymer	Excellent electrical conductivity, combine with the substrate material easily	Flexible circuit
	Carbon nanobases	Adsorption, conductivity, thermal conductivity, low mass, high specific surface area, degradability	Large area flexible circuit

#### 3.1.1 Substrate/packaging materials

The substrate material is required to have high scalability and flexibility to ensure that the flexible wearable sensor is soft and deformable. Common substrate materials include polydimethylsiloxane (PDMS), Eco-flex, polyurethane (PU), aerogel, hydrogel and polyurethane (PI). PDMS is a silicone rubber elastic polymer and widely used as the substrate material of flexible wearable sensor due to the excellent optical transparency, high stretchability. However, this material has poor conductivity, so scholars have introduced conductive materials for preparation of flexible sensors (Jin et al., 2020), (Li et al., 2020a). Eco-flex has good stretchability, which can be used to fabricate crop flexible sensors with large stretch effect due to excellent stretchability. Zhang et al. (2022a) prepared a self-winding flexible electrode that can be transformed from two-dimensional to three-dimensional by using the difference in elastic modulus between PDMS and Eco-flex, which can be used for non-destructive monitoring of plant stem flow. PU has excellent tensile strength and tear strength. Liang et al. (2021) used PU as the substrate, and Graphene (Gr) was quickly implanted into the surface of the molten PU fiber to successfully prepare a humidity-responsive flexible sensor. When the Gr implantation amount was 4.1%, the elongation at break of the PU-based humidity-sensitive fiber sensor was 91.5%, which showed excellent flexibility. Aerogel is a nano-porous solid material formed by replacing the liquid phase in the gel with a certain drying method by sol-gel method. Yang et al. (2023) prepared a robust super elastic spider web-like conductive composite aerogel by freeze-drying and thermal imidization technology. As a pressure sensor, the aerogel has a wide linear sensing range between 0.01 kPa and 53.34 kPa, ultra-low detection limit, high sensitivity, fast response/recovery time (85/80 ms) and compression response (500 mm·min^-1^) and excellent resistance to cyclic fatigue. Hydrogel is an advanced material that can adjust the moisture content and film properties by changing the three-dimensional cross-linked structure. In the process of transition from liquid to solid, a variety of flexible films can be formed (Yuk et al., 2019). Hsu et al. (2021) used acrylic acid, reduced graphene oxide and polyaniline as reaction materials to prepare a hydrogel with a double network structure. Using the deformable properties of the hydrogel, the flexible sensor realizes the monitoring of plant growth and ammonia concentration. Chen et al. (2023) used polyacrylamide-acrylic acid as the base hydrogel, and obtained PPCH hydrogel by Fe^3+^ oxidative polymerization after single-sided infiltration of aniline. The conductivity, mechanical and strain sensing properties of PPCH hydrogel were mainly investigated.

As the most studied flexible substrate, PI was used as a sensor protective layer to prevent external stimuli from affecting device performance due to the excellent heat resistance and environmental stability (Garland et al., 2018). In addition, substrate materials with good transparency and air permeability and adhesion are selected to optimize the biocompatibility and wearability of the flexible sensor. Im et al. (2018) directly adhered wearable sensors to the surface of crop leaves to avoid damage caused by clamping and fixing. In order to increase adhesion, a single-sided viscous polyethylene terephthalate (PET) film was added to PI as a substrate. PET film has single-layer adhesion, which can make the sensor adhere to the back of the blade well and effectively eliminate the external force damage caused by the clamp (Qu et al., 2021).

Packaging material is the outermost layer of flexible electronic devices, which is wrapped with functional circuits inside and contacts with the application environment outside. Flexibility, ductility, waterproof, transparency transmittance and electromagnetic shielding of packaging material were required. At present, most of the packaging materials for flexible sensors were flexible polymer films that are the same as the substrate materials, such as PI/PDMS/PET. Among them, PI is the most common choice for crop wearable packaging materials because of their transparency and durability. In the encapsulation of water-oxygen-insensitive circuits, the material can be modified by flexible polymer layering to achieve the encapsulation. Jeong et al. (2019) used a chemical vapor deposition method to plate a layer of 200 nm thick Poly-p-dichlorotoluene as an encapsulation layer, thereby transforming the polyvinyl alcoho (PVA) surface into a hydrophobic surface, which improved the measurement error caused by the hydrophilicity of the substrate material PVA of the blade humidity sensor. In the future, with the widespread application of flexible sensors in field measurement, the demand for packaging materials will be more diversified.

#### 3.1.2 Functional materials

The functional materials of flexible sensor include conductive materials and semiconductor materials such as metal films, liquid metals, flexible conductive polymers, and carbon nanobases. As functional materials for crop flexible sensors, metal nanomaterials are often dispersed in solution to prepare conductive inks, and transferred to flexible substrates by printing and coating (Kalia et al., 2020). Liquid metal is a kind of liquid metal at room temperature, which not only can conduct electricity, but also has lower viscosity, excellent surface tension and biological safety (Jiang et al., 2022). Flexible conductive polymer refers to a polymer material with conductive properties, which has excellent conductivity and combine with substrate materials easily. Meder et al. (2021) used PEDOT to prepare flexible electrodes for plant electrical signal monitoring. Kim et al. (2020) applied CI-doped PEDOT to plant surface to monitor plant ultraviolet radiation damage. Carbon-based nanomaterials, represented by carbon nanotubes and graphene, have high electrical conductivity and excellent bending properties of material monomers. Graphene oxide (GO) is widely used in plant flexible humidity sensors due to the unique 2D structure and super permeability to water molecules (Lv et al., 2019).

Semiconductor materials can be divided into inorganic semiconductor materials and organic semiconductor materials. Inorganic semiconductor materials need to be designed or thinned to achieve flexibility. Organic semiconductors are organic materials with semiconductor properties, which are flexible and prepared by sputtering, evaporation, sol-gel, printing and other methods. Min et al. (2021) used a simple all-solution process to fabricate a hydrophobic, stretchable carbon nanotube implanted conductive composite electrode on the insect bionic adhesive structure, and developed a beetle-inspired adhesive patch. The patch had a nanocomposite structure and was suitable for isotropic stretchable flexible electronic skin.

### 3.2 Fabrications of flexible electronics

In addition to the selection of flexible materials, the preparation of flexible sensors mainly includes the preparation process of sensing layers and thin films (Qu et al., 2021). The preparation of sensing layers refers to the integration of conductive sensing materials with flexible bases. Common preparation methods (Yin et al., 2021) include lithography (Yang et al., 2021), printing (Kang et al., 2020), and roll-to-roll manufacturing (Lee et al., 2022). Lithography is one of the core technologies to realize the complex microstructure of integrated circuits. Dadras-Toussi et al. (2022) fabricated enzyme-based flexible biosensors on PDMS substrates by lithography process, which can be used for various microelectronic components and devices. This process can also be used in the preparation of crop sensors due to the high resolution, which can achieve high-precision complex microstructures and optimize the accuracy and performance of sensors. Common printing methods include soft etching (Ikumapayi et al., 2021), nano-imprinting (Cox et al., 2020), screen printing (Gong et al., 2022) and jet printing (Li et al., 2021a). The screen printing method is simple in principle, low in cost, and suitable for large-area printing. However, the sensor prepared by screen printing has low resolution, only tens of microns, far less than lithography technology. With the development of printing technology, more printing technologies (Lemarchand et al., 2022) mentioned in **Table 2** have been applied to the preparation of sensing layers. According to the actual situation of product index, ink performance, production conditions and market positioning, appropriate technology can be selected to realize pattern design. The roll-to-roll manufacturing technology has the characteristics of large scale, high yield and low cost, which is suitable for large-scale production and manufacturing of crop flexible electronics, which is beneficial to promote the layout of crop flexible electronic network and the expansion of application fields.

**Table 2:** Requirements and characteristics of printing technologies for sensing layers.

Technique	Lateral resolution (μm)	Ink viscosity (cP)	Adapting substrate	Film thickness (nm)	Line width (μm)	Print speed (mm/s)
Screen-printing	30–100	1000–50000	Polymer, paper, plastic, glass	14000–25000	40	50–300
Gravure	5–20	20–500	Plastic, paper	10–400	35	5–1000
Flexography	80	50–500	Plastic, Polymer	5–50	3	200–830
Offset	10–50	40000–100000	Plastic	0.5–2.5	10	200–800
Inkjet-printing	20–50	1–30	Polymer, paper, plastic, glass, fabric	100–500	2–20	1.25–7000
Aerosol	10	1–1000	Polymer, plastic, glass	30–150	10	0.1–10

The technology of preparing thin films mainly includes spin coating, sputtering deposition, evaporation deposition, sol-gel method, chemical vapor deposition. Spin coating is the most commonly used and simplest method for preparing plant flexible electronic films (Chai et al., 2021). The sputtering deposition method refers to the process in which atoms and molecules in the target material are overflowed and deposited to form a thin film by bombardment of high-energy particles in a relatively vacuum environment. The preparation of conductive layer by sputtering deposition is a common processing technology for plant flexible sensors. The conductive layer material of the sensor prepared by this method is dense and uniform, and has high adhesion to the substrate. Chemical vapor deposition is characterized by low deposition temperature, easy control of film composition and good uniformity. At present, many studies have applied chemical vapor deposition to prepare “electronic tattoos” on plant surfaces to monitor plant physiological information (Kim and Andrew, 2020; Park et al., 2016).

## 4 Application of flexible wearable sensor in crop monitoring

Flexible wearable sensors have shown great potential in the agriculture field due to their excellent extensibility, biocompatibility, and long-term real-time monitoring. Flexible electronic devices were used in crop monitoring and fixed on crop tissues and organs in combination with various wearing methods to monitor crop phenotypes and growth environment information in real time. This section summarizes the application scenarios of flexible wearable sensors in crop monitoring, including crop nutrient, physiological, ecological information and growth environment monitoring.

### 4.1 Detection of crop nutrient information

#### 4.1.1 Nutrient substance

**Figure 3:**
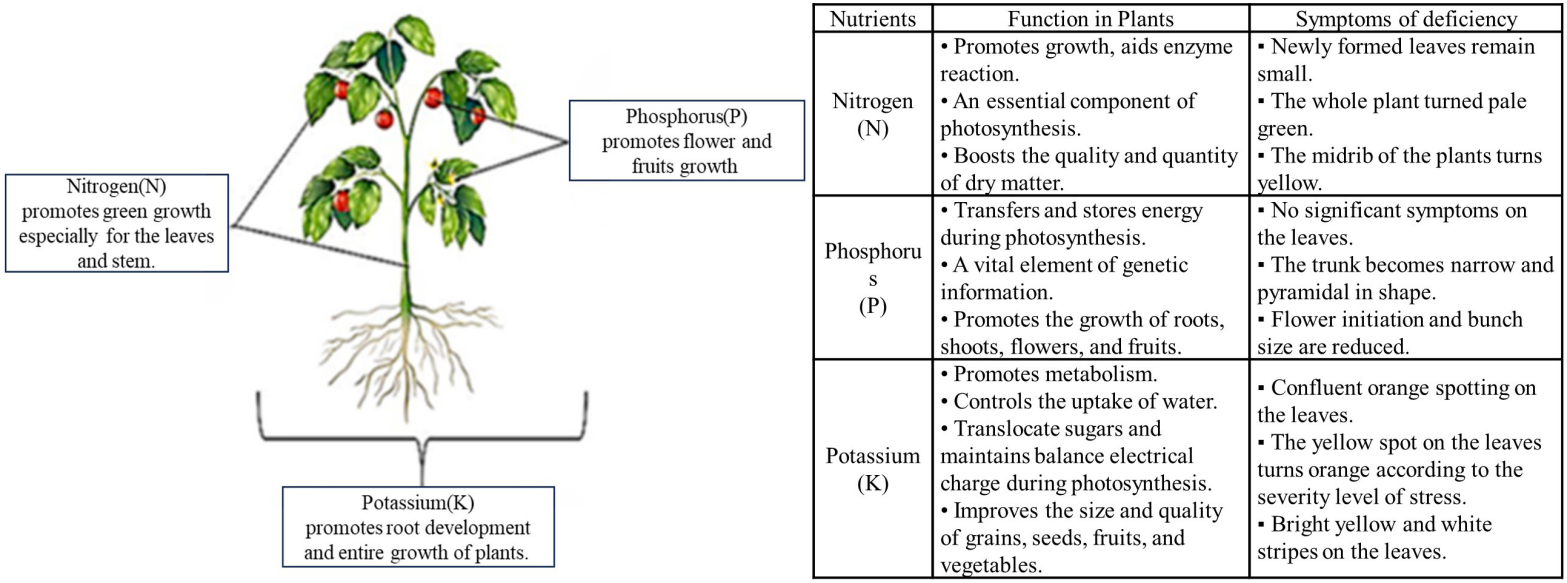
Function and stress symptoms of N, P and K in crops.

Nutrients such as nitrogen (N), phosphorus (P) and potassium (K) play an important role in the leaves, flowers, fruits and roots of the crop. They are essential substances for crop growth and development, yield and quality. As shown in **Figure 3**, N is the primary factor limiting crop growth and yield formation. It is an essential substance for crops to produce protein and promote tissue development. When N is deficient, the synthesis of chlorophyll, protein, enzyme and other compounds in plants is affected to inhibit photosynthesis of crop (Kim et al., 2020). P is a structural component of nucleic acids in crop genetic information, which contributes to the transfer and storage of energy during photosynthesis (Nam et al., 2006). K improves crop disease resistance by inducing changes in tissue metabolites and reducing xylem vessel water potential, and regulates water and sugar intake during photosynthesis (Malhotra et al., 2018). In addition, the real-time monitoring of glucose, chlorophyll and other information provides important data support for the healthy growth of crops.

Nagamine et al. (2023) demonstrated the feasibility of extracting a potassium ion (K^+^), which was one of the most important electrolytes in plant cell physiology, using the leaching phenomenon. Additionally, a wearable K^+^ sensor film was placed in contact with the leaf surface via a membrane soaked with a phosphate-buffered saline (PBS)-based extraction solution to show the minimally invasive extraction and continuous monitoring of K^+^ in a plant leaf for the first time. Diacci et al. (2021) used a minimally invasive organic electrochemical transistor sensor to monitor the glucose content in the phloem of the plant stem. The concentration of metabolites in the plant were monitored in real time by this minimally invasive sensor. The physiological response of the wound was very small enough to affect the overall metabolite concentration in the plant, but the long-term measurement reliability of the sensor was affected during the healing process. Chen et al. (2024) present an electrochemical sensing device that combines microneedle sensors and 3D printing technology, continuous monitoring for 12h and salt stress treat on tomato and aloe vera were performed to verify the feasibility of integrated device applied to real-time glucose detection in plants. Church et al. (2018) proposed a solid contact microion selective electrode, which successfully explored the zinc ion transport process in citrus leaves and roots by monitoring the changes of electrical signals. It should be noted that the wounds caused by such invasive detection may still affect the long-term measurement reliability of the crop sensor during the healing process.

Zhang et al. (2023b) designed a miniatured, flexible, and wearable chlorophyll meter capable of *in situ*, long-term plant monitoring, which was inspired by these reflectance-based indices. It employed a monochromatic LED and a pair of symmetric PDs for incident radiation and measurement of the intensity of the reflected light. The chlorophyll content was calculated based on the relationship between leaf chlorophyll content and spectral reflectance. This meter was 1.5 mm thick and weighed 0.2 g, making it 1000 times lighter than the commercial chlorophyll meter. It could be patched onto the upper epidermis of the leaf tightly and realized long-term monitoring with little negative impact on leaves and plants. Li et al. (2021b) designed a wearable sensor for real-time analysis of VOC markers in plant volatile organic compounds. The sensor was based on a multifunctional reduced graphene oxide (rGO) chemical resistance sensor array, which formed reversible interactions with various plant volatile organic compounds through hydrogen bonds or halogen bonds. The rGO nanosheets are modified with functionalized gold nanoparticles (AuNPs) or directly modified with chemical ligands containing different recognition groups to selectively capture nitrogen-containing organic compounds from various plant volatile organic compounds. Flexible wearable devices show great application potential in crop nutrient monitoring. Combined with biomarkers (Hwang et al., 2012), they can be used to monitor plant nutrient absorption and transport in real time, which is of great significance for studying crop growth and nutrient stress.

#### 4.1.2 Water content

The nutrient information can be extended to crop water content. The proportion of water quality in crops is about 80%-90%, which is a key factor affecting crop growth. Accurate control of water content is essential for crop management. Flexible wearable sensors were used to monitor the changes of leaf water content and leaf surface humidity, which judged the water status of crops and provided data support for accurate irrigation (Atherton et al., 2012).

**Figure 4:**
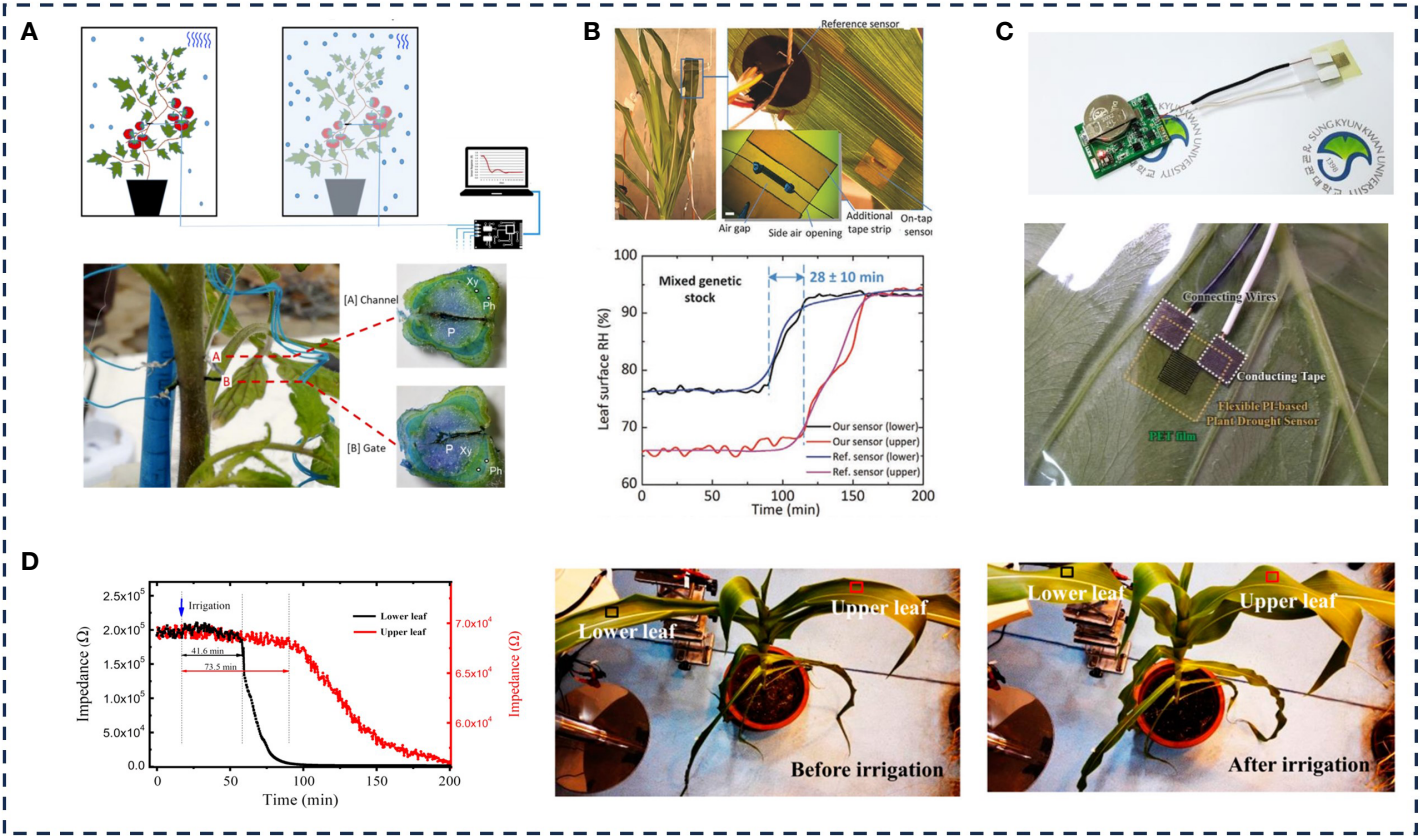
Application examples of water content sensors.

Vapor pressure deficit (VPD) refers to the difference between saturated water vapor pressure and actual water vapor pressure at a certain temperature, which can indirectly reflect the stomatal opening and closing of crops and further determine the water use efficiency of crops (Yuan et al., 2019). When the VPD is too low, the water molecules in the crop are saturated and condensation occurs. When the VPD is too high, the water vapor pressure difference is too large, which causes the plant to die due to the loss of the mass water. Vurro et al. (2019) designed an invasive sensor based on organic electrochemical transistor to monitor VPD in plants. A regular relationship between the resistance value measured between the two electrodes and VPD was established, and the plant water consumption was obtained through resistance change (**Figure 4A**). Due to the serious damage of invasive sensors to crops, non-invasive sensors can be used to estimate VPD by monitoring the change of crop surface humidity. Oren et al. (2017) innovatively designed a tape-based wearable sensor (**Figure 4B**) using graphene as a functional material, achieving a patterned graphene sheet resistance of 0.22 ± 0.12kΩ sq^-1^. By tracking the change of leaf relative humidity, the time required for the internal moisture of maize to reach the target leaf through the xylem from the root was successfully estimated.

Besides resistance-type moisture sensors, capacitance-type and impedance-type moisture sensors have also been applied in crop hydration monitoring. Im et al. (2018) developed a flexible plant drought sensor based on PI, which used water evaporation to monitor plant stomatal response. The drought sensor was formed by depositing Ti/Au electrodes on the flexible PI film, which is both a sensing element and a supporting substrate. Then the sensor is stripped from the glass and transferred to a highly flexible single-sided viscous polyethylene terephthalate film, which helps to attach it well to the leaves without causing strain or tension. It can be integrated with the readout processing circuit and the Bluetooth Low Energy (BLE) module to obtain the tobacco water status in real time (**Figure 4C**). The experimental results showed that the sensor has good sensitivity to humidity changes. When the relative humidity increases from 55% to 90%, the capacitance value increases from 2.72 pF to 206 pF. However, PET tape caused water vapor to accumulate between the sensor and the leaf surface, which had an impact on the stability of the sensor. Kim et al. (2020) used vapor printing technology to prepare chlorine-doped PEDOT electrodes for monitoring blade capacitance and impedance signals. Different with other adhesive films, the vapor-printed polymer electrode was not stratified from the living surface of the micro-texture with biological maturity. Plant drought and ultraviolet light damage were monitored within 130 days by analyzing bioimpedance spectroscopy. The results showed that the water content of leaves and the capacitance value were decreased with the aggravation of drought. Lan et al. (2020) used graphene oxide (GO) as the humidity-sensitive material to obtain a flexible capacitive-type GO-based humidity sensor with low hysteresis, high sensitivity, and long-term stability. The flexible humidity sensor can be directly attached onto the plant leaves for real-time and long-term tracking transpiration from the stomata, without causing any damage to plants. Peng et al. (2024) proposed the wearable electrodes for real-time monitoring of leaf capacitance. Gold nanoparticles were magnetron sputterred on PET membrane to form the wearable Au@PET electrodes, which could be conformally attached to the leaf surface to form leaf capacitance sensor. It was found that capacitance value was positively correlated with leaf moisture content.

In addition, crop sap flow is also an important physiological indicator of water consumption, nutrient distribution and crop health (Miner et al., 2017). The transpiration rate of branches or tillers can be determined by measuring plant sap flow rate. Li et al. (2022) proposed a new type of moisture sensor based on flexible graphene oxide (GO). The sensor had high sensitivity, fast response and good biocompatibility. The humidity monitoring sensitivity reached 7945W/% RH, and the response time was 20.3s, which can dynamically monitor the internal water migration of plants (**Figure 4D**).

When using a flexible wearable sensor to measure crop moisture, the interface between the sensor and the crop must be strong to accurately obtain moisture information. The measurement principle can only be used for most herbaceous crops, but cannot be used for fruit trees with thick bark, which limits the application range of the sensor.

### 4.2 Detection of crop physiological information

Crops grow in outdoor environments for a long time, and are often subjected to abiotic and biotic stresses. Therefore, monitoring crop stress response and taking timely intervention measures have very important significance. At present, stress conditions and health status of crop are often analyzed by monitoring electrical signals or chemical substances.

#### 4.2.1 Crop electrical signals

Electrical signals are essential for regulating crop life activities, and their changes can reflect the internal physiological information of crops. When crops response to external stimuli, the membrane potential changes rapidly (Senavirathna and Muhetaer, 2020).

**Figure 5:**
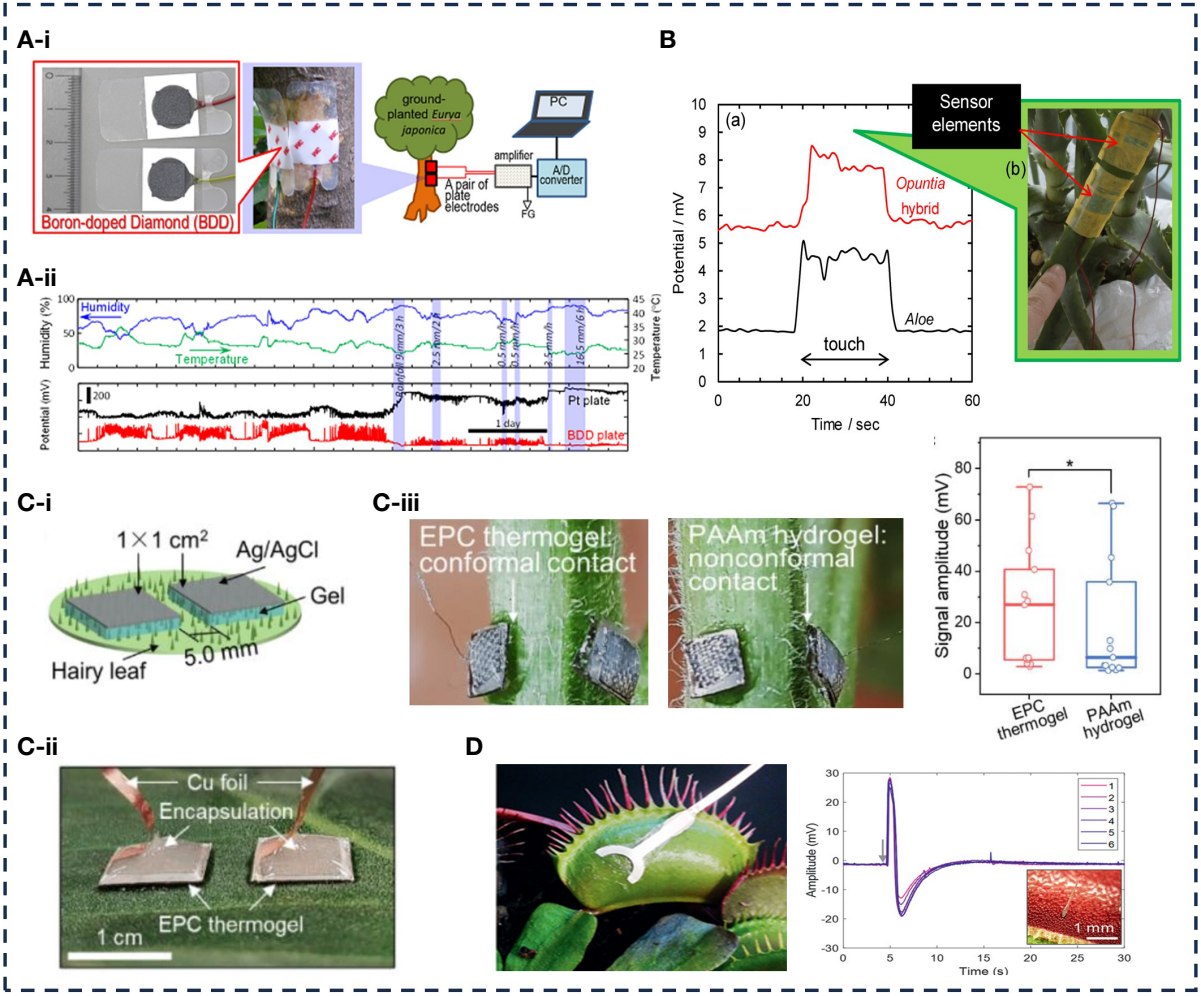
Application examples of crop electrical signal sensors.

The flexible wearable sensor can effectively avoid the data distortion caused by the wound while capturing the changes of electrical signals such as potential, impedance and capacitance. Ochiai et al. (2015) proposed a sensitive plant monitoring system for monitoring biopotentials (**Figures 5A-i**). The boron-doped diamond (BDD) electrode sensor was connected to the green phloem tissue to monitor the biopotential in the plant. When the finger touched the cactus mixed surface or when environmental factors such as temperature and humidity changed, the BDD sensor detected significant changes in biopotentials (**Figures 5A-ii**). Compared with Pt or Ag electrodes, the sensitivity of BDD electrode is 4–7 times higher to the change of biopotential of potted cactus hybrid plants, and BDD electrode can monitor continuously and effectively the change of biopotential for a long time. Tago et al. (2017) developed a composite sensor based on BDD powder and resin, which was covered with a masking tape to prevent electromagnetic noise interference. Attached to the surface of aloe leaves, the biopotential changes caused by rainfall or finger touch were successfully monitored (**Figure 5B**). Compared with the pure BDD sensor, the BDD/resin composite sensor showed higher signal stability. In order to accurately monitor bioelectrical signals, it is necessary to ensure that the flexible electrode is closely integrated with the blade. However, the epidermal structure of different crops is different according to different crop physiology, which makes the flexible electrode attached to the surface of the crop different. The above studies are only for smooth skin plants such as aloe and cactus, which are not suitable for hairy surfaces, which may hinder the detection of biopotential.

For hairy plants, Luo et al. (2021) prepared a non-invasive deformable ion electrode based on thermal gel. Using the liquid-solid deformation of the thermal gel, the sensor was tightly adhered to the surface of the hairy plant, and the electrical signal fluctuation of the plant under fire and mechanical damage was successfully detected. The thermal gel polymer was a multi-block amphiphilic copolymer composed of hydrophilic polyethylene glycol (PEG), thermally responsive polypropylene glycol (PPG) and hydrophobic biodegradable polycaprolactone (PCL) segments as shown in **Figure 5C-i**. When the temperature raised, the PPG segment was dehydrated, prompting the formation of supramolecular hydrogel matrix in the relevant micelle structure. A metal plate was placed on the electrode assembly completed by the thermogel to establish adhesion and shape preservation on the hairy plant. The electrode had a certain mechanical strength and electrical interface, and recorded high-fidelity electrophysiological signals (**Figure 5C-ii**). Based on the flame-induced potential changes of EPC thermogel and polyacrylamide (PAAM) hydrogel electrodes in hairy sunflowers, the median voltage signal of EPC thermogel was significantly higher than that of PAAM hydrogel. The voltage signal of PAAM hydrogel was mainly concentrated at about 10mV, which was much lower than that of EPC thermogel (**Figure 5C-iii**). This kind of electrode used a high concentration of ionic conductive gel to increase the adhesion and conductivity of the electrode, which would cause damage to the crop. Meder et al. (2021) reported a plant flexible self-adhesive electrode and successfully tracked the electrical signal changes of the fly grass plant (**Figure 5D**). The electrode was flexible and ultra-thin, and adhered to the surface of different plants only by van der Waals force.

#### 4.2.2 Crop volatile organic compounds (VOCs)

Since crops can exchange information with the external environment by releasing VOCs (Holopainen and Gershenzon, 2010) (Kegge and Pierik, 2010), different VOCs will be released when crops are subjected to mechanical damage, insect feeding activities, pathogen infection, drought, extreme temperature and other abiotic stresses. According to this feature, crop flexible sensors can monitor plant physiological conditions in real time by capturing concentration changes of VOCs to achieve disease prevention and stress warning (Lew et al., 2020).

Most of the previously reported VOCs detection methods for plant disease diagnosis rely on commercially available devices such as gas chromatography-mass spectrometry (GC-MS) (Pelo et al., 2021) and electronic noses (Karakaya et al., 2019). However, GC-MS method requires complex gas sampling processes, intensive labor force, expensive instruments, and skilled operators, thus making it not suitable for in-field measurement. Unlike GC-MS, the electronic nose captures overall information about the VOCs, generating “fingerprint data” instead of qualitative and quantitative results for the measured sample. This acquired data is then trained to differentiate between various VOCs features in the sample’s headspace through appropriate tuning (Gan et al., 2023).

**Figure 6:**
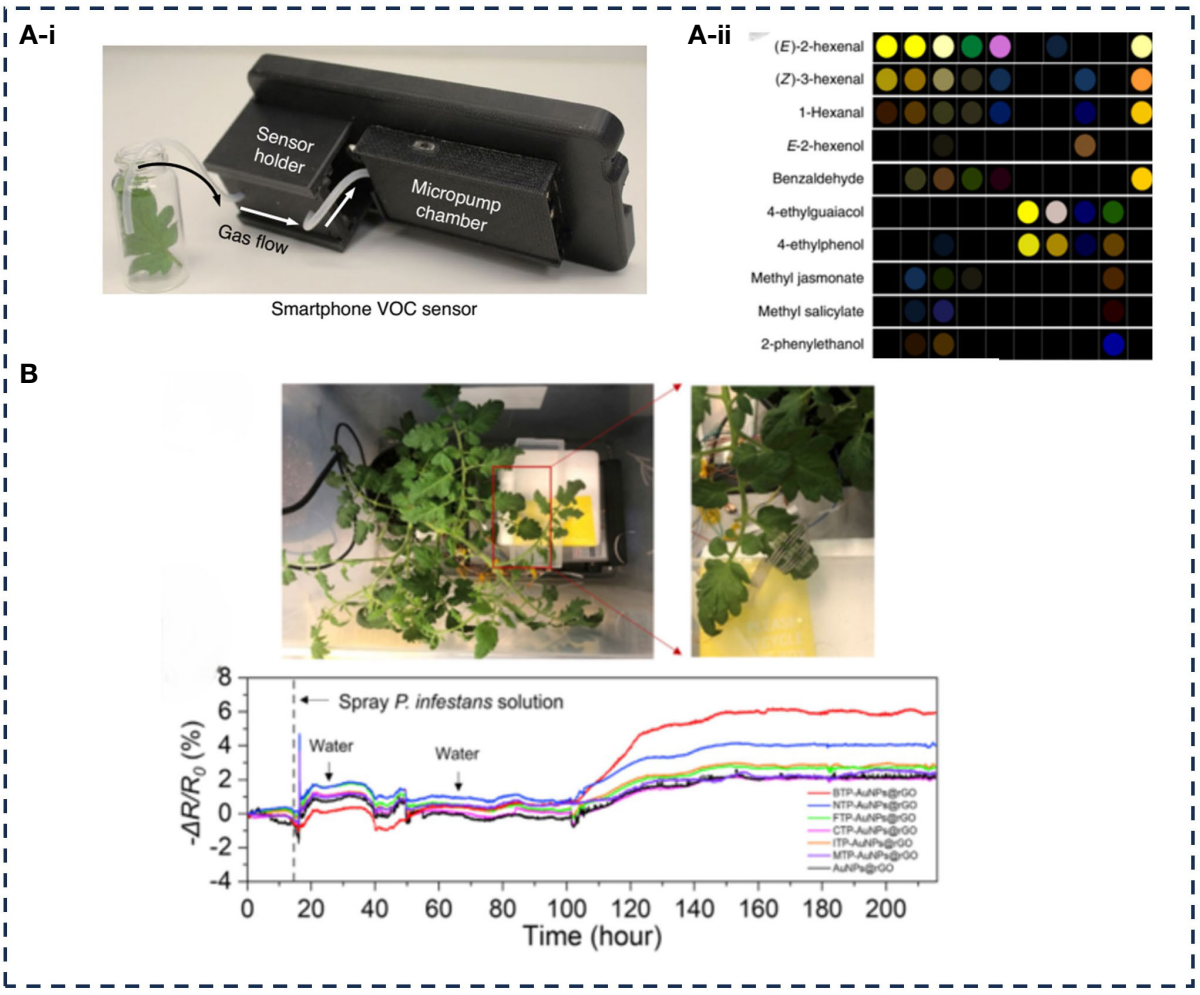
Application examples of VOCs monitoring.

Given the growing demand for long-term and real-time monitoring of plant symptoms, wearable sensors with continuous and noninvasive monitoring of VOCs are emerging, which need to be small. Li et al. (2019) successfully diagnosed plant diseases by monitoring the volatiles of leaves. The paper-based colorimetric sensor array was integrated with a mobile phone to design a VOC volatile handheld device (**Figure 6A-i**). By monitoring the VOC emissions of field leaves, non-invasive diagnosis of early late blight was achieved (**Figure 6A-ii**). The device integrated a disposable colorimetric sensor array composed of plasma nanocolorants and chemical-responsive organic dyes, which can detect key plant volatiles within 1 min, and the recognition accuracy reached 10^–6^. The sensor array can change the color according to different VOCs, to identify 10 common plant volatiles, which can be used to monitor tomato late blight. Based on the research results, the team developed a flexible sensor for real-time monitoring of plant VOC (**Figure 6B**) (Li et al., 2021b). This sensor can identify 13 different plant VOC, which was used to monitor the health status and stress status of plants. Ibrahim et al. (2022) reported a wearable plant sensor that can monitor methanol emission directly on the leaf of a plant under field conditions with low cost, high portability, and easy installation and use. The wearable device was fabricated using flexible materials, poly (ethylene terephthalate), which allowed it to conformably adhere to the surface of the plant’s leaves. The sensing component of the device was constructed by depositing poly (2-amino-1, 3,4-thiadiazole) and catalytic platinum nanoparticles (PtNPs) on the surface of a carbon electrode using cyclic voltammetry. This configuration enabled efficient electrochemical oxidation of methanol at a specific potential, resulting in high sensitivity and selectivity. The device demonstrated the capability to detect methanol concentrations at sub-parts ppm levels. Although plant VOCs monitoring sensors have good development prospects, the stability and selectivity of the technology in dealing with different pressure sources and pathogen infections still was be studied.

Compared with the traditional detection methods using large instruments in the laboratory, using plant flexible sensors to monitor crop electrical signals and volatile organic compounds in real time has advantages in terms of monitoring efficiency, monitoring accuracy, and detection indicators.

### 4.3 Detection of crop ecological information

Quantitative and accurate measurement of crop growth is the basis for understanding the regulation mechanism of crop growth. Crop growth is a highly dynamic process (Cosgrove, 2018). Real-time monitoring of crop canopy height (Allen and Pereira, 2009), leaf length (Tripler et al., 2011), fruit growth rate and other ecological information is of great significance for improving crop yield (Zhen et al., 2017). Therefore, choosing the appropriate measurement tools is necessary. At present, there are some methods for measuring crop growth, such as optical phenotype method, which can realize non-invasive continuous monitoring of crop growth, but there is a problem of low monitoring accuracy due to the occlusion of the light path by other branches or leaves (Hartmann et al., 2011). Flexible sensing technology provided a new idea for improving the monitoring accuracy of crop ecological information.

**Figure 7:**
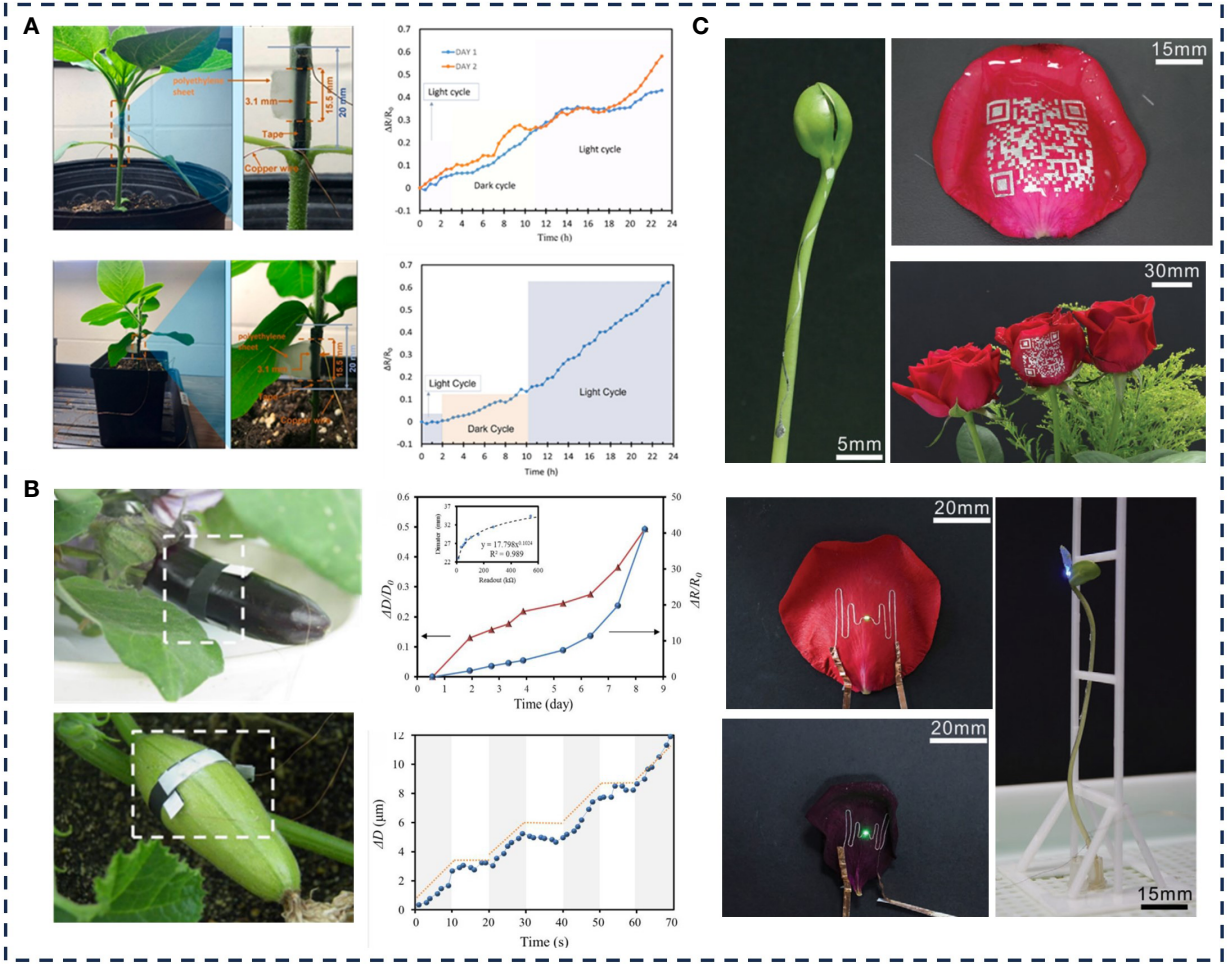
Application examples of crop ecological information monitoring.

Borode et al. (2023) developed a polyaniline (PANI)/elastic band strain sensor for real-time measurement of plant growth. The sensor used the dip-coating method to realize the *in-situ* chemical polymerization of aniline and the elastic band substrate. The polyaniline nanoparticles were combined with the substrate fiber and its gap to make it conductive, and the elastic band substrate was stretched to make it stretchable. By connecting the sensor to the stem internodes of sunflower and soybean, the early growth monitoring of sunflower and soybean plants was realized in real time. The measurement results showed that the sunflower mainly grows in the dark cycle, and the growth was almost stable in the light cycle. The ΔR/R0 accumulated by the PANI strain sensor corresponded to an increase of about 267μm on the first day and 355μm on the second day, or an average of 311μm per day, with a total increase of 622μm (**Figure 7A**). The total ΔR/R0 of the strain sensor for soybean accumulation within 24 hours was 0.6, corresponding to a growth of about 373μm. The results were consistent with the fluctuating growth pattern of sunflower and soybean in Reference (Perilli et al., 2012) and (Dobrescu et al., 2017). Tang et al. (2019) fabricated a flexible, stretchable and wearable carbon nanotube/graphite sensor by synergistic enhancement between graphite and carbon nanotube (CNT) films, and simply depositing graphite ink, CNT ink and curing, which can evaluate plant growth from nanometer to centimeter level. The growth of eggplant and gourd was measured in real time by the sensor and self-made readout circuit integration device to capture that the fruit of eggplant and gourd had a rhythmic growth pattern (**Figure 7B**). Jiang et al. (2020) used gallium-based liquid alloy (LA) with high fluidity and conductivity to fabricate a strain sensor with 200% high stretchability, which was directly printed on the epidermis of roses and bean sprouts to monitor the water content and length of leaves (**Figure 7C**).

Although the above sensors can detect plant growth changes, the detection range of plant growth is limited due to the material itself. To solve this problem, Hsu et al. (2021) used plant flexible sensors prepared by hydrogel materials to give a solution. The tensile strain range of the sensor can reach up to 200% by applying hydrogel materials to customize the shape.

### 4.4 Detection of growth environment information

The growth and physiological processes of crops are affected by fluctuations in growth environments such as soil conditions, climate change, and growth temperatures (Hackenberger et al., 2018). Therefore, monitoring crop growth environment parameters, such as temperature, humidity, light, gas, etc., and understanding the interaction between crops and their growth environment can more effectively maintain crop health and increase crop yield.

**Figure 8:**
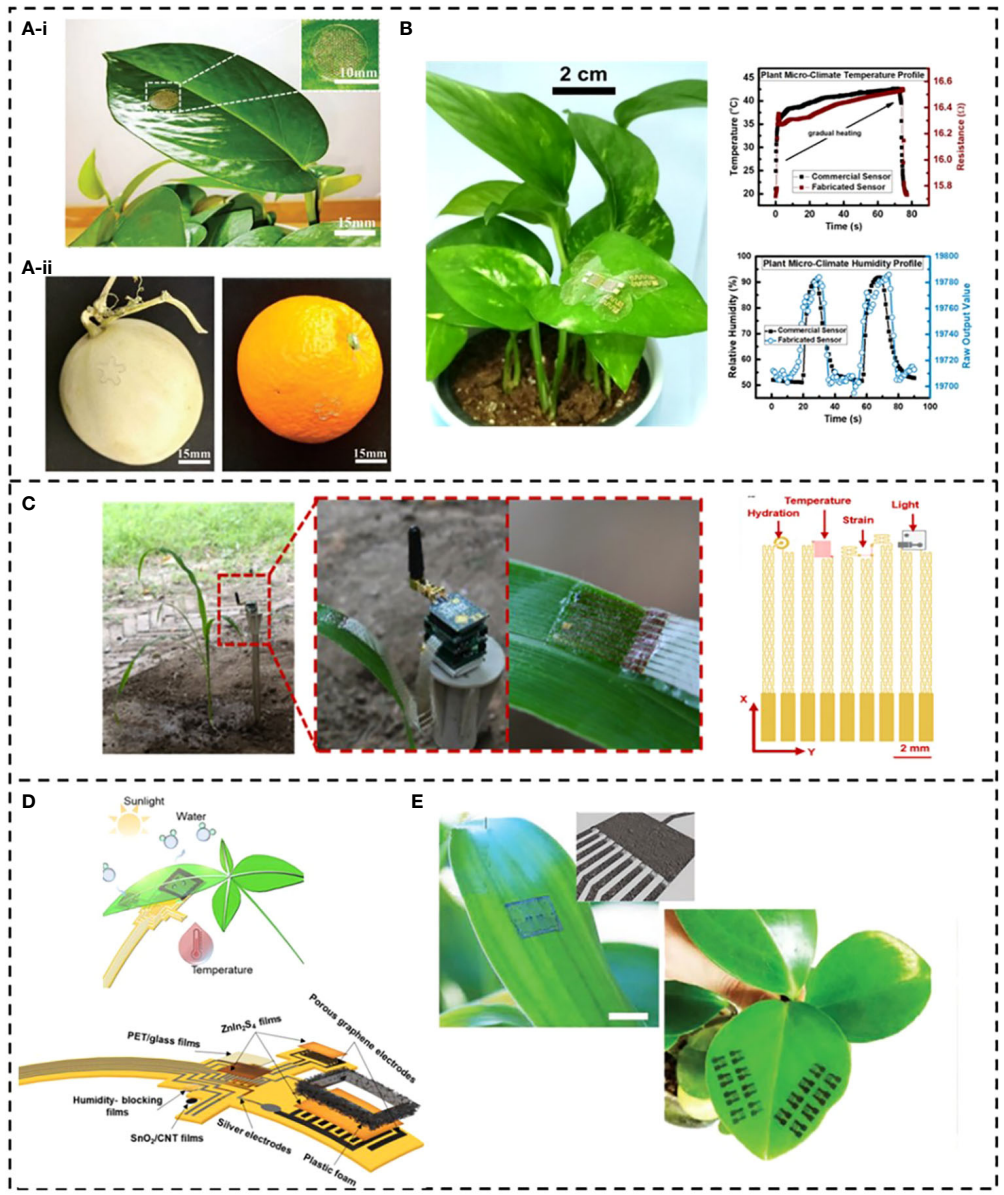
Application examples of crop growth environment information monitoring.

Crop flexible climate sensors are attached to the surface of crop organs to sense the target climate in real time, avoiding the lag of monitoring methods such as spectroscopy (Mulla, 2013), machine vision (McCarthy et al., 2010), and aerial vehicles (Primicerio et al., 2012). Dong et al. (2023) developed a flexible temperature sensor with porous matrix for leaves and fruits, which can minimize the influence of the sensor on plant respiration and provide channels for various volatile organic compounds (VOC), CO_2_, O_2_ and water vapor to ensure normal physiological activities of leaves. After 20 days of integration on plant leaves, the sensor can continuously monitor the microenvironment temperature for 24 hours without causing dysfunction due to growth (**Figure 8A-i**). The fruit temperature sensor is designed to meet the structure of its surface characteristics, especially the surface of the same scale as the sensor, to solve the problem of shape conversion from plane to three-dimensional. The strain generated during the stretching process is small, and it can work on various complex surfaces without losing its performance (**Figure 8A-ii**). *In vitro* experimental results show that these two sensors are expected to monitor the microenvironment temperature in plant biology.

In recent years, sensors have gradually developed from single monitoring to diversification to have stronger market competitiveness. Flexible multi-functional sensors that can monitor simultaneously crop physiology and environmental parameters have also appeared in agriculture field. Nassar et al. (2018) developed a wearable device that integrates temperature and humidity sensors that can be deployed on plant surfaces to monitor plant microclimate and growth rate (**Figure 8B**). Zhao et al. (2019) designed an ultra-light, ultra-thin, stretchable plant sensor that can continuously monitor light, temperature, humidity, and plant growth at the same time (**Figure 8C**), which helps to understand the growth of plants in different environments. The snake-shaped island bridge was designed to optimize the stress distribution, and the ultra-thin elastic substrate PDMS was used to reduce the tensile constraint of the sensor. In terms of temperature detection, the sensor achieved a temperature resolution of 0.2°C, in terms of monitoring plant growth, the sensor monitored 15.5% leaf elongation in the vertical ground direction, and the signal-to-noise ratio of the sensor was less than 3% when the multi-function operates simultaneously. However, the overall fabrication process of the sensor is relatively complex, which requires multiple magnetrons sputtering and oxygen plasma etching to achieve multi-functional simultaneous operation. Lu et al. (2020) used stacked ZnIn_2_S_4_ nanosheets as the core sensing medium to prepare a multi-mode flexible sensing system for plant growth management, which can achieve continuous monitoring for up to 15 days and guide the transpiration and stomatal opening and closing state of plants (**Figure 8D**). The sensing system consisted of two humidity sensors, a temperature sensor and an illumination sensor. These sensors were integrated on a flexible PI film with a thickness of 50um. Screen-printed silver paste and laser-induced graphene were used as interconnecting electrodes. In order to distinguish light and humidity perception, the PET film was covered on the illumination sensor to isolate water interference. The illumination and humidity sensor electrode were designed as a planar interdigital structure, which realized humidity sensing range up to 90%, and high-sensitivity monitoring of light response within 4ms at high frequency.

In addition, toxic gases in the environment can also cause irreversible damage to crops, such as NH_3_, CO_2_ and O_3_ (Kumar et al., 2017). Li et al. (2020b) designed a flexible sensor based on mixed polyaniline/ti_3_c_2_t_x_ sensitive film for monitoring NH_3_ around plants. Considering the influence of humidity and temperature on gas measurement, a spray gas sensor array was used to detect toxic gases in a real-time and wireless manner by directly printing on plant leaves. The flexible sensor had good adaptability on planes and surfaces (**Figure 8E**). The results showed that the flexible NO_2_ gas sensor had higher sensing ability and lower detection limit, and its sensitivity was even much higher than that of rigid sensing materials.

The multi-functional flexible sensor can meet the diversified monitoring of crop growth environment, and provide accurate and valuable evaluation basis for agricultural managers to comprehensively evaluate crop growth status. However, the volume and quality of the sensor have increased due to the limitations of preparation materials and processes, to bring burden to crops. In the future development, the lightweight and miniaturization of multifunctional flexible electronics are an important development direction.

## 5 Challenges and perspectives

The rapid development of intelligent agriculture puts forward higher requirements for the accuracy and biosafety of crop sensors. Flexible sensing technology can monitor crops growth status and external environmental fluctuations in real time, which is of great significance for improving crop yield. However, most of the existing flexible wearable devices are suitable for laboratories. In practical applications, the large number of crops and the complex growth environment are great challenges for flexible sensors. New crops wearable sensing devices need to be developed to improve the stability and reliability of crop flexible wearable sensing in complex environments. In solving the practical application problems of crop monitoring, there are still a lot of work to be studied, such as expanding the application scenarios of flexible wearable devices in crop monitoring, manufacturing materials with high biocompatibility, developing reliable self-powered modules and multi-functional crop monitoring systems, which need further research:

(1) Researching new sensing technology and expanding the application of flexible sensors in crop monitoring. At present, crop wearable sensors mostly convert crop phenotype and environmental information into electrical signals, which in turn reflect crop growth status and stress conditions. However, the crop physiological information expressed by electrical signals is limited, such as the key indicator of nitrogen content cannot be monitored. Due to the different internal substances of crops, their spectral information and sound signals are different. Therefore, optical and acoustic devices can be integrated into wearable sensors (Li et al., 2023), (Wang et al., 2023) to obtain more signal types. It provides more reliable theoretical guidance for crop physiology research.
(2) Developing new preparation materials to reduce the negative impact of crop sensors on crop growth and development. The flexible sensor is covered on the surface of the leaf, or attached to the crop stem, which will affect the growth and development of the crop. The growth rate of plants is affected by electrodes, which may reduce crop photosynthesis and respiration, and affect nutrients of leaf. When working in the field, the leakage of sensor chemicals will also cause pollution to the soil environment. Therefore, measures such as optimizing conductive materials and optimizing electrospinning process are taken to improve the transparency of the electrode to minimize interference and achieve lightweight, soft and stretchable crop wearable sensors. The breathable waterproof material is designed based on micro-nano technology (Zhang et al., 2022b), which will not affect the gas exchange of crops after bonding with crops, while protecting electronic devices from external environmental pollution. The sensor can be prepared by selecting biodegradable materials with good ductility and biocompatibility to avoid polluting farmland.
(3) Designing new sensing structures to solve the mismatch problem caused by the rapid growth of flexible sensors for crops. In addition to selecting materials with good tensile properties and low elastic modulus, the flexibility of the sensor can also be greatly improved through two-dimensional or three-dimensional structures, such as waves, folds, island bridge structures, self-similar structures, origami, cracks and interlocks. At present, inspired by the biological characteristics of nature, bionic methods were used to design sensor structures. For example, self-similar snake-shaped wires can be prepared on pre-stretched flexible substrate materials, or the substrate is designed to be honeycomb or serrated, so that the snake-shaped wire is partially suspended to minimize the constraints of the flexible substrate on the wire.
(4) Researching on new power supply technology to supply energy for crop flexible sensing system. The power supply mode of plant flexible sensors greatly restricts the application scenarios of plant flexible electronic devices. Traditional rigid batteries hinder the development of compactness and compatibility of flexible sensor systems. Miniaturized environmental energy harvesters are integrated, such as triboelectric nanogenerators, flexible solar photovoltaic devices, and flexible piezoelectric films. Converting the energy around the sensor into available electricity to meet the requirements of crop wearable sensing systems for small-volume batteries, strong endurance, and flexible energy modules. In addition, the development of wireless power transmission methods is considered, including near-field and far-field radio frequency (RF) technology, near-field technology based on inductive coupling and magnetic resonance, and far-field technology based on radiation power transmission, which have power transmission and data communication functions, and can reduce sensor quality and reduce maintenance. However, the near-field technology can only be realized within a limited distance, and there are strict requirements for emitter-receiver alignment. Far-field technology can cover a large area, but due to the omnidirectionality, its power transmission efficiency is low, and it is vulnerable to obstruction interference.
(5) Building a crop sensor network to realize multi-parameter large-scale monitoring of field crops. Crop flexible sensors have excellent characteristics such as high flexibility, stretchability, transparency, light weight, and high resolution. Integrating them into crop leaves or stems can only obtain local crop phenotypes or environmental information, and cannot monitor the overall phenotype and environmental information of host crops, and other crop information in the same field. Multiple flexible sensors are applied, and multi-functional and multi-dimensional agricultural electronic sensor networks are built to expand the monitoring coverage and realize wireless monitoring of large-scale farmland crop information. Combining with automated agricultural machinery equipment such as automatic sprinkler irrigation and automatic harvesting, the unmanned management of large-scale intelligent farms is realized.

## 6 Conclusions

With the continuous advancement of agricultural modernization and intelligence, crop flexible sensors become an indispensable application equipment, which is expected to completely change crop production and management. By providing real-time data on crop health and environmental conditions, agricultural productivity is optimized and the impact of agriculture on the environment is minimized to feed a growing global population. At the same time, the distributed sensor network is expected to draw a large area of crop growth map and obtain the overall situation of farm. The efficient management of agricultural production and resources is realized by analyzing crop growth and environmental data. With the further development of material science and intelligent sensing, crop flexible sensors will be further developed and more widely used in the future.

## Author contributions

BY: Conceptualization, Investigation, Methodology, Software, Writing – original draft. FZ: Conceptualization, Funding acquisition, Project administration, Resources, Supervision, Writing – review & editing. MW: Data curation, Methodology, Software, Visualization, Writing – review & editing. YZ: Data curation, Validation, Writing – review & editing. SF: Funding acquisition, Resources, Supervision, Writing – review & editing.
